# Mitochondrial Metabolism as Target of the Neuroprotective Role of Erythropoietin in Parkinson’s Disease

**DOI:** 10.3390/antiox10010121

**Published:** 2021-01-15

**Authors:** Federica Rey, Sara Ottolenghi, Toniella Giallongo, Alice Balsari, Carla Martinelli, Robert Rey, Raffaele Allevi, Anna Maria Di Giulio, Gian Vincenzo Zuccotti, Serena Mazzucchelli, Roberta Foresti, Michele Samaja, Stephana Carelli

**Affiliations:** 1 Laboratory of Pharmacology, Department of Health Sciences, University of Milan, Via Antonio di Rudinì 8, 20142 Milano, Italy; federica.rey@unimi.it (F.R.); toniella.giallongo@unimi.it (T.G.); alice.balsari@studenti.unimi.it (A.B.); annamaria.digiulio@unimi.it (A.M.D.G.); 2 Pediatric Clinical Research Center Fondazione “Romeo ed Enrica Invernizzi”, Department of Biomedical and Clinical Sciences, University of Milan, 20157 Milan, Italy; raffaele.allevi@unimi.it (R.A.); gianvincenzo.zuccotti@unimi.it (G.V.Z.); serena.mazzucchelli@unimi.it (S.M.); 3 Laboratory of Biochemistry, Department of Health Sciences, University of Milan, Via Antonio di Rudinì 8, 20142 Milano, Italy; sara.ottolenghi@unimi.it; 4 Laboratory of Pathological Anatomy, Department of Health Sciences, University of Milan, Via Antonio di Rudinì 8, 20142 Milano, Italy; carla.martinelli@unimi.it (C.M.); robert.rey@unimi.it (R.R.); 5 Department of Pediatrics, Children’s Hospital “V. Buzzi”, 20154 Milan, Italy; 6 University Paris Est Creteil, INSERM, IMRB, F-94010 Créteil, France; roberta.foresti@inserm.fr

**Keywords:** erythropoietin (EPO), redox imbalance, Parkinson’s disease, mitochondrial metabolism, neuroprotection, tyrosine hydroxylase (TH)

## Abstract

Existing therapies for Parkinson’s disease (PD) are only symptomatic. As erythropoietin (EPO) is emerging for its benefits in neurodegenerative diseases, here, we test the protective effect driven by EPO in in vitro (SH-SY5Y cells challenged by MPP^+^) and in vivo (C57BL/6J mice administered with MPTP) PD models. EPO restores cell viability in both protective and restorative layouts, enhancing the dopaminergic recovery. Specifically, EPO rescues the PD-induced damage to mitochondria, as shown by transmission electron microscopy, Mitotracker assay and PINK1 expression. Moreover, EPO promotes a rescue of mitochondrial respiration while markedly enhancing the glycolytic rate, as shown by the augmented extracellular acidification rate, contributing to elevated ATP levels in MPP^+^-challenged cells. In PD mice, EPO intrastriatal infusion markedly improves the outcome of behavioral tests. This is associated with the rescue of dopaminergic markers and decreased neuroinflammation. This study demonstrates cellular and functional recovery following EPO treatment, likely mediated by the 37 Kda isoform of the EPO-receptor. We report for the first time, that EPO-neuroprotection is exerted through restoring ATP levels by accelerating the glycolytic rate. In conclusion, the redox imbalance and neuroinflammation associated with PD may be successfully treated by EPO.

## 1. Introduction

Parkinson’s Disease (PD) is the second most common neurodegenerative disorder, after Alzheimer’s Disease [1]. The main histopathological hallmarks of PD are Lewy bodies, constituted mainly by alpha synuclein aggregates, which increase oxidative stress, leading to cell toxicity, mitochondrial disfunction, neuronal apoptosis and subsequent neuroinflammation [2,3,4]. By affecting the Substantia Nigra, these mechanisms drive dopaminergic neurons degeneration and typical movement impairments [5,6].

Erythropoietin (EPO), a type I cytokine essential for erythroid development and maturation [7,8], is also produced in the CNS [9,10,11], where it exerts neurotrophic and neuroprotective actions [12,13,14]. In recent years, four different isoforms of EPO receptor (EPOR) have been found in the brain [14,15,16,17,18] that likely mediate different neuroprotective pathways [14,15,16,19]. These isoforms include the canonical 55 Kda EPOR isoform, a heterodimer formed by the interaction with the beta common receptor βcR CD131, a truncated isoform found in the dopaminergic neurons of the CNS, and a soluble version of the receptor found in murine brains [16,17,18,20]. 

Numerous cell studies, both in animals and humans, have shown that the administration of recombinant human EPO (rhEPO) might be effective against hypoxic, ischemic, and traumatic brain injury, as well as chronic and progressive degenerative diseases [16,21,22,23]. Interestingly, studies have reported rhEPO’s ability to counteract such key processes that are altered in PD [24,25,26,27,28,29,30,31]. The neuroprotective role of rhEPO has been investigated in vivo mainly in rodent models of PD induced by rotenone, 1-Methyl-4-phenyl-1,2,3,6-tetrahydropyridine (MPTP) [32,33] or 6-hydroxydopamine (6-OHDA) [34]. In these studies, rhEPO has been shown to restore the level of tyrosine hydroxylase (TH) and to decrease the level of tumor necrosis factor alpha (TNFα) [34,35]. Two phase I clinical trials have also been performed in PD patients, showing no adverse effects for rhEPO [25,36]. Specifically, in one PD trial, rhEPO administration (40,000IU each; i.v. twice a week for 5 weeks) ameliorated nonmotor symptoms 12 months after administration [25]. The hypothesis that EPO may be an effective neurotrophic and neuroprotective molecule originates from observations obtained with EPO-releasing neural precursors cells (Er-NPCs) transplantation in an animal experimental model of PD obtained by MPTP administration [37,38]. First, Er-NPCs, which produce and release EPO physiologically, show high rates of neuronal differentiation that depend on autocrine EPO release [39]. Second, Er-NPCs also have a high therapeutic potential following their transplantation both in experimental PD and traumatic spinal cord injury preclinical models [37,38,40]. Many pieces of evidence also suggest that the neuroprotective effects of EPO are related to increased resistance to oxidative stress and stabilization of the redox equilibrium, for example, through the Janus kinase-signal transducer and activator of transcription pathway and the up-regulation of anti-apoptotic genes, as observed in vitro and ex-vivo [41,42]. Moreover, it has also been reported that EPO can promote synaptogenesis, neurite repair and spine formation [43,44].

In this study, we aim to dissect the mechanism of action of EPO in both an in vitro and an in vivo consolidated PD experimental model [38,40,45]. We demonstrate that EPO plays a pivotal role in counteracting the loss of the dopaminergic phenotype in SH-SY5Y cells intoxicated with 1-methyl-4-phenylpyridinium (MPP^+^). In this context, we report that EPO prevents the alterations induced by MPP^+^ in mitochondrial morphology and fuels the glycolytic process to compensate for the loss of activity of mitochondrial complex I. We also show the therapeutic efficacy of EPO in a murine model of PD, describing that EPO improves the behavioral outcome and the recovery of dopaminergic markers. 

## 2. Materials and Methods

### 2.1. SH-SY5Y Cells Culture

SH-SY5Y cells are widely used for the study of neurodegenerative diseases, and represent an ideal in vitro model for PD studies [45]. SH-SY5Y cells were grown in DMEM-F12 medium supplemented with 10% Fetal Bovine Serum (Euroclone, Pero, Milano, Italy), 1% glutamine (Gibco, ThermoFisher Scientific, Waltham, MA, USA) and 1% penicillin/streptomycin (Gibco, ThermoFisher Scientific). Cells were split every 4–5 days after trypsinization (trypsin-EDTA 1X 0.05% ThermoFisher Scientific). Cells were subjected to 3 experimental conditions: non-treated (NT), treated with 500 μM MPP^+^ (MPP^+^), and treated with 500 μM MPP^+^+4U/mL EPO (MPP^+^+ EPO). MPP^+^ iodide (Sigma-Aldrich, Merck Life Science, Milano, Italy) was freshly weighed for each experiment, dissolved in PBS, diluted in the appropriate medium and administered for 24 or 48 h. Recombinant human EPO (rhEPO) was obtained from Johnson & Johnson (Eprex™, New Brunswick, NJ, USA).

### 2.2. MTT Assay

Cell viability, measured by a quantitative colorimetric MTT assay sensitive for the cell metabolic status, reflects early redox changes. Briefly, SH-SY5Y cells were seeded in a 96-well plate at a 2 × 10^5^ cells/well density. At the end of the treatments (24 h or 48 h), 10 μL MTT assay kit reagent (Sigma-Aldrich, Saint Louis, MO, USA) was added to each well, and cells were incubated for 3 h. MTT crystals were eluted with 100 μL of elution solution, composed of 4 mM HCl, 0.1% (*v*/*v*) NP40 all in isopropanol for 30 min. The relative absorbance was measured with EnSight™ multimode plate reader (PerkinElmer, Waltham (HQ), MA, USA) at λ = 560 nm. The results are expressed as a percentage of the absorbance read in NT cells.

### 2.3. Immunocytochemistry

SH-SY5Y cells were seeded on ethanol-washed glass coverslips, maintained in the appropriate culture medium, and then processed for immunocytochemistry following a described protocol [39]. Briefly, cells were fixed with 4% paraformaldehyde in 0.1 M PBS (Life Technologies, Thermo Fisher Scientific, Waltham, MA, USA), pH 7.4, for 20 min at room temperature, and then washed with PBS. The coverslips were incubated overnight at 4 °C in PBS containing 10% normal goat serum (NGS, ThermoFisher Scientific), 0.3% Triton X-100 (BDH, VWR, Radnor, PA, USA), and the appropriate primary antibody. The cell characteristics were assessed by immunocytochemistry with the following antibodies: anti-erythropoietin receptor (EPOR; 1:200; Genetex, Irvine, CA, USA), and anti-TH (1:500; Sigma-Aldrich, Saint Louis, MO, USA). Cells were thoroughly rinsed with PBS and 10% NGS and reacted with the appropriate secondary antibody (Alexa Fluor^®^ 488 and 546, Life Technologies) for 1.5 h. Nuclei were stained with DAPI at the final concentration of 1 µg/mL for 10 min. Glass coverslips were mounted using the FluorSave Reagent (Calbiochem, Merck Chemical, Darmstadt, Germany) and analyzed by confocal microscopy (Confocal laser scanning microscopy platform Leica TCS SP8, Leica Microsystems). As the control, the appropriate secondary antibody was administrated omitting the primary one (Alexa Fluor^®^ 488 or 546, Life Technologies). To favor visibility, the brightness of all immunochemistry images was multiplied by 3, except for the TH immunocytochemistry, for which the brightness was multiplied by 4. The correction was applied by Adobe Photoshop 2020 to the entire image, without altering data appearance. 

### 2.4. RNA Extraction and Real Time PCR

Total RNA was extracted using TRIZOL^®^ reagent (Life Technologies, Carlsbad, CA, USA) following the manufacturer’s instructions, and then quantified (NANOPhotometer^®^ NP80, IMPLEN). Total RNA (1 μg) was reverse transcribed using iScript cDNA synthesis kit (Bio-Rad, Hercules, CA, USA) according to the manufacturer’s instructions. Real-Time PCR was performed with StepOnePlus^TM^ Real-Time RT-PCR System (Thermo Fisher, Waltham, MA, USA) using SsoAdvanced^TM^ Universal SYBR ^®^ Green Supermix (Bio-Rad). The NCBI’s Primer-BLAST tool was used to design primers, which are listed in Supplementary Table S1. Gene expression was calculated using the 2^−ΔΔCt^ method. GAPDH was used as endogenous control.

### 2.5. Western Blot

Cell protein extracts were obtained by means of RadioImmunoPrecipitation Assay (RIPA) lysis buffer. Proteins were quantified with the Bradford Assay following standard protocol (Coomassie Plus—The Better Bradford Assay^TM^ Reagent, Thermo Scientific). Equal amounts of solubilized proteins were heated in Laemmli sample buffer (Bio-Rad) containing 70 mM 2-β-mercaptoethanol (Sigma Aldrich, Saint Louis, MO, USA), separated by SDS-PAGE gel 10% and electroblotted onto a nitrocellulose membrane (GE Healthcare, Amersham, Chicago, IL, USA). Membranes were then blocked in 5% slim milk (diluted in TBS with 0.05% Tween-20) and probed with the appropriate primary antibody: monoclonal anti-TH; 1:5000; Sigma-Aldrich, T2928), polyclonal anti-PINK1 (1:1000; Genetex, GTX107851), monoclonal anti-β-actin 1:1000; Sigma-Aldrich, A5441), monoclonal anti-GAPDH (GAPDH 1:1000; Cell Signaling, #2118), polyclonalanti-erythropoietin receptor (EPOR; 1:200; Genetex, GTX37704), overnight at 4 °C. The EPOR antibody targets both the canonical 55 kDa isoform and the 37 kDa isoform (Supplementary Figure S1). The membrane was then incubated with specific secondary antibody Peroxidase AffiniPure Goat Anti-Rabbit/Mouse IgG (1:10,000 dilution; Jackson Immuno Research, Cambridge, UK). Proteins were visualized by mean of an enhanced chemiluminescence detection system (ECL™, Amersham, Chigago, IL, USA). After acquisition by a GelDoc^TM^ image capture system (Kodak, Rochester, NY, USA), the proteins present on the nitrocellulose membrane were quantified using ImageJ software.

### 2.6. Mitotracker Analysis

This technique enables marking mitochondria with the fluorescent molecule Mitotracker Red CMXRos (Invitrogen, Thermo Fisher Scientific, Waltham, MA, USA). After treatment, the medium was removed, and cells were incubated for 30 min with 100 nM MitoTracker reagent. Cells were rinsed three times with PBS and fixed with 4% paraformaldehyde for 15 min. Nuclei were stained with DAPI (1µg/mL final concentration, 10 min at room temperature), mounted using the FluorSave Reagent (Calbiochem) and analyzed by confocal microscopy (Confocal laser scanning microscopy platform Leica TCS SP8, Leica, Heidelberg, Germany). Please see above for acquisition procedure. 

### 2.7. Transmission Electron Microscopy (TEM) and Quantification

Cells gently scraped from the culture flasks were fixed in 2.5% glutaraldehyde in 0.13 M phosphate buffer, pH 7.2–7.4, for 2 h, post-fixed in 1% (*w/v*) osmium tetroxide, dehydrated through graded ethanol and propylene oxide, and embedded in epoxy resin. Several semithin sections were prepared from each sample and stained with 0.5% toluidine blue in 1% sodium borate. Ultrathin sections of 50 to 60 nm were counterstained with uranyl acetate and lead citrate, to be observed using a Tecnai Spirit BT transmission electron microscope (Thermo Fisher Scientific, Waltham, MA, USA). The cytoplasmic area (nuclei excluded) was measured on 18,000× printed micrographs (previously converted in digital bitmap images by Image Processing and Analysis in Java-ImageJ) in 8 areas at a fixed 4 mm distance from one to another according to the Marquez simplified method [46]. Briefly, the number of degenerated mitochondria was counted in 8 randomly selected areas. Finally, the ratio between the number of degenerated mitochondria per area unit (=100 µm^2^) was calculated. The mean number of 8 areas was considered as the final value for each group.

### 2.8. Cell Respiration and Glycolytic Process

The acute effect of MPP^+^ and the potential beneficial action of EPO on SH-SY5Y cell metabolism were assessed using the Seahorse Bioscience XF24 Extracellular Flux Analyzer (XF24, Agilent, Santa Clara, CA, USA). This technique measures the oxygen consumption rate (OCR) and the extracellular acidification rate (ECAR, an index of glycolysis) in real time in viable adherent cells. SH-SY5Y cells were seeded on the specific Seahorse XF24 cell culture plate (Agilent, Santa Clara, CA, USA) at an 8 × 10^5^ cells/well density 24 h before the assay, as described previously [47]. The day of the assay, the growth medium was replaced with an assay medium containing 5.5 mM glucose, 1 mM pyruvic acid, 1 mM L-glutamine, according to the manufacturer’s instructions. After three basal measures, OCR and ECAR were measured for 200 min in the different experimental conditions. 

In a separate experiment, OCR and ECAR were measured 24 h after treatment. A Mitostress assay was performed to assess mitochondrial function. The test consists of the sequential injection of the following modulators of the mitochondrial respiratory chain: oligomycin (1 µg/mL), an inhibitor of ATP synthase and ATP linked mitochondrial respiration, carbonyl cyanide-4-(trifluoromethoxy)phenylhydrazone (FCCP, 1 µM; Sigma-Aldrich, Saint Louis, MO, USA), an uncoupler of mitochondrial respiration, and rotenone/antimycin A (R/AA, 1 µM; Sigma-Aldrich, Saint Louis, MO, USA), inhibitors of complex I and complex III, respectively, that cause a complete impairment of mitochondrial function [47]. This test allows the quantification of different parameters, including basal respiration, ATP-linked respiration, proton leak, maximal respiratory capacity and non-mitochondrial respiration [48,49]. 

### 2.9. ATP Assay

The ATP levels were measured in cells 4 and 24 h after treatment with the different experimental conditions using the ATPlite Luminescence Assay System (PerkinElmer). Cells were seeded the day before the assay in a 96-well plate (80,000 cells/well) and the assay was performed according to the manufacturer’s instructions.

### 2.10. Animals and Study Approval

For this study, we used adult C57BL/6J male mice (Charles River, Calco, Lecco, Italy), 12–15 weeks old and weighing 20 to 24 g. Before the experiments were carried out, the animals were kept for at least 7 days in standard conditions (22 ± 2 °C, 65% humidity, and artificial light between 08:00 a.m. and 08:00 p.m.) with ad libitum food and water supply. Mice were trained for 1 week before being treated with 1-methyl-4-phenyl-1,2,3,6-tetrahydropyridine (MPTP, the prodrug of MPP^+^; Sigma-Aldrich, Saint Louis, MO, USA) in order to acclimatize them to behavioral testing. The procedures were performed according to the Italian Guidelines for Laboratory Animals, in total respect of the European Communities Directive of September 2010 (2010/63/UE). The study was approved by the Review Committee of the University of Milano (N° 778/2017).

### 2.11. Induction of Parkinsonism, Treatments and Groups of Animals

12 weeks old C57BL/6J mice were subjected to a first intraperitoneal injection of MPTP (36 mg/kg). Motor dysfunction was evaluated every 2 days for the following 10 days. Then the animals were subjected to a second injection of MPTP (20 mg/kg) [37,38]. Three days after the second MPTP injection, the animals were infused with recombinant human erythropoietin (rh-EPO) (#100-64; Peprotech, London, UK) at a dosage of 1U/g of animal body weight (*n* = 6) [37,38]. rh-EPO was infused in the striatum according to the following stereotaxic coordinates in relation to bregma: 0.1 mm posterior, 2.4 mm mediolateral and 3.6 mm dorsal at the level of left striatum [50]. For this reason, in the present study, rh-EPO was administered in the same site as Er-NPCs in the previous one. This choice would further validate EPO’s therapeutic role highlighting its effects at the striatum level. The infusion rate of EPO was 1 µL/min (total 5 µL). After the infusion, animals were monitored for 24 h, subjected to antibiotic therapy (Gentamicin 1mg/mL, SIGMA) and rehydrated with saline solution. Experimental animals were divided into three groups: (1) control not-treated (NT, healthy animals, *n* = 6), (2) MPTP-treated mice (MPTP, *n* = 6), (3) MPTP-treated mice infused with recombinant human erythropoietin (MPTP+ rh-EPO; *n* = 6).

### 2.12. Behavioral Tests

To investigate the recovery of motor dysfunction after rh-EPO infusion, two behavioral tests were performed: horizontal and vertical grid tests [37,38]. Each animal was tested twice at each time point. The analysis was performed by three observers in blind.

### 2.13. Horizontal Grid Tests

The grid apparatus was built following the model developed by Tillerson and co-workers [37,38,51]. During the test the animal was recorded for 30 s and the recordings were replayed in order to assess the percentage of forepaw faults using the slow-motion option. The number of unsuccessful forepaw steps in relation to the total number of attempted forepaw steps was assessed [51]. Mice were acclimatized to the grid twice a day for 1 week, before MPTP treatment.

### 2.14. Vertical Grid Tests

The vertical grid apparatus was built following the model developed by Kim and co-workers [52]. For this test, the mouse was put 3 cm away from the top of the apparatus, facing upwards, and it was recorded when turning around and climbing down. The score assessed refers to the time required by the mouse to turn around, climb down, and reach the bottom of the grid with its forepaw, all within 180 s [37,38,52]. Mice were acclimatized to the grid twice a day for 1 week before MPTP treatment.

### 2.15. Sacrifice and Brain Dissection

Three animals for each group were anesthetized with an intraperitoneal injection with sodium pentobarbital (65 mg/kg of body weight), subsequently perfused through the left ventricle with 50 mL of saline solution and lastly fixed with 200 mL of 4% paraformaldehyde in 0.1 mol/L PBS. The brains were removed and cryoprotected at 4 °C in sucrose 300 g/L in 0.1 mol/L PBS solution for further analyses and sectioning.

### 2.16. Immunohistochemistry and Quantitative Analysis 

Immunohistochemistry analyses were performed on 20 μm coronal sections of the whole brain, cut at −25 °C using a cryostat (Leica). Both ipsilateral and contralateral striatum were analyzed, with no measured fluorescence intensity differences. Specimens were transferred onto glass slides, rinsed with PBS and treated with blocking solution (10% NGS, 0.2% Triton X-100), as described in refs. [37,38]. The following primary antibodies were used: anti-Glial Fibrillary Acidic Protein (GFAP; 1:1000; Covance, Princeton, NJ, USA; #PRB-571C), anti-Nuclear Receptor Related-1 Protein (NURR1; 1:1000; Abcam, Cambridge, UK; #ab41917), anti-Tyrosine Hydroxylase (TH, 1:500; Sigma Aldrich, #T2928), anti-ionized calcium-binding adapter molecule 1 (IBA1) (1:250; Abcam, #ab178680); anti-MOuse MAcrophage (MOMA)/CD68 (1:250; Millipore, Burlington, MT, USA; #MAB1852). The following secondary antibodies were used: Alexa fluor 543 goat-anti-mouse IgG (1:1000; Invitrogen, Life Technologies, Carlsbad, CA, USA), Alexa fluor 543 goat-anti-rabbit IgG (1:1000; Invitrogen, Life Technologies). Images were acquired in the region corresponding to bregma 2.80/3.52 mm as indicated in the Paxinos and Franklin atlas [53] and analyzed with a confocal laser scanning microscopy platform (Leica TCS SP8, Leica). In control experiments, primary antibodies were omitted, and only the appropriate secondary antibody was administered (Alexa Fluor^®^ 488 or 546, Life Technologies, Carlsbad, CA, USA). All the analyzed sections had the same immunostaining conditions, with same staining solutions. The microscope light intensity of the laser was the same for all the analyses and for determining the background optical density. ImageJ (NIH) software was used for microphotographic digital analysis [37,38,40,54,55] assessing the number of positive pixels against the negative background [37,38,40] in three slides (2 images/slides) from three animals per condition (*n* = 9). For the cells quantification, the number of positive cells was counted with respect to the total number of nuclei.

### 2.17. Statistical Analysis

Statistical evaluations were completed using GraphPad Prism 7.0a version (GraphPad Software Inc, La Jolla, CA, USA). For the MTT in vitro assays, one-way ANOVA was used followed by Tukey’s post-test. In the other in vitro assays and animal immunohistochemistry, one-way ANOVA was used followed by Dunnett’s post-test. For all in vitro experiments, data are reported as mean±SEM. Behavioral data are expressed as mean±SD and analyzed with two-way ANOVA followed by the Bonferroni’s post-test. Repeated measures ANOVA tests with time and group (NT, MPTP, MPTP+EPO) as factors were applied. The level of statistical significance was set at *p* = 0.05.

## 3. Results

### 3.1. EPO Restores Cell Viability in an in Vitro Model of PD

**Figure 1:**
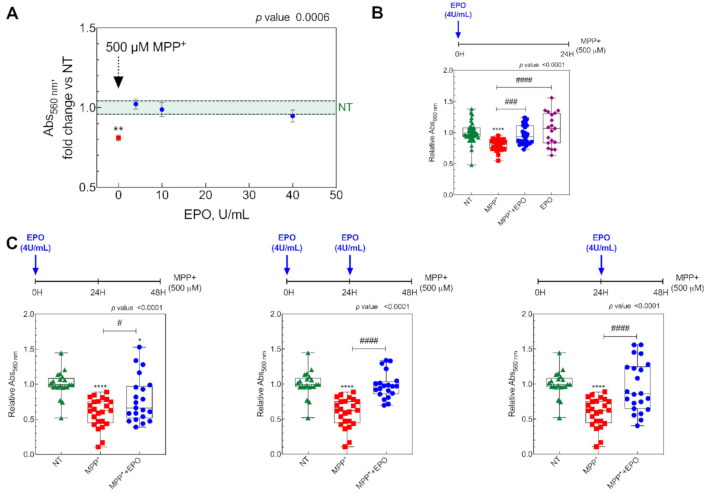
In vitro results showing the effect of erythropoietin (EPO) on cell viability. (**A**) Cell viability in SH-SY5Y cells treated with 500 µM MPP^+^ (MPP^+^), and MPP^+^ plus 4, 10 and 40 U/mL EPO for 24 h. Data are expressed as mean ± SEM of 8 replicate values in 3 independent experiments (*n* = 24) and results are represented as percent of non-treated (NT), shown as the light green area. ** *p* < 0.01 vs. NT. (**B**) Cell viability in SH-SY5Y cells (NT), cells treated with 500 µM MPP^+^ (MPP^+^), cells treated 500 µM MPP^+^ + EPO 4U/mL (MPP^+^ + EPO) and cells treated with 4U/mL EPO (EPO). Data are expressed as box-and-whisker plot of 8 replicate values in 3 independent experiments (*n* = 24). Data are represented as a fraction over non-treated (NT). **** *p* < 0.0001 vs. NT; ### *p* < 0.001, #### *p* < 0.0001 vs. MPP^+^. (**C**) Effect of 4U/mL EPO on cell viability during 48 h MPP^+^ treatment. Viability was evaluated after 48 h in the three different experimental conditions in which EPO was administered at three different time points as reported in the timeline above each graph. Data are expressed as box-and-whisker plot of 8 replicate values in 3 independent experiments (*n* = 24) and results are represented as fraction over NT values. * *p* < 0.05, **** *p* < 0.0001 vs. NT; # *p* < 0.05, #### *p* < 0.0001 vs. MPP^+^.

To obtain an in vitro model of PD, SH-SY5Y cells (5 × 105 cells/cm^2^) were treated with 500 µM MPP^+^ and kept at 37 °C under 5% CO2. The protective effect of EPO for 24 h was then evaluated at three concentrations (4, 10 and 40 U/mL) by assessing cell viability by the MTT assay (Figure 1A). As expected, MPP^+^ reduced cell viability, but co-treatment with EPO rescued viability at all the concentrations (Figure 1A). As all concentrations elicited similar effects, all further experiments were run at 4 U/mL EPO, the minimal concentration with full effects. Indeed, when looking at EPO’s effect on MPP^+^-treated cells, the EPO4 dosage was sufficient to restore viability, whereas EPO only-administration did not affect cellular viability (Figure 1B).

Next, we aimed at testing the persistence of the effects of EPO to discriminate if this effect is protective or restorative. To this purpose, SH-SY5Y cells were treated with MPP^+^ for 48 h and the protective effect of one or two 4 U/ML EPO administrations was tested in various experimental schemes, as shown in Figure 1C. Administration of 4 U/mL EPO resulted in rescued cell viability in all conditions (Figure 1). For the subsequent analyses, SH-SY5Y cells were exposed to three conditions: NT, 500 µM MPP^+^, and 500 µM MPP^+^ + 4 U/mL EPO for 24 h.

### 3.2. EPO Administration Promotes PD-Specific Phenotypic Recovery in Vitro 

**Figure 2:**
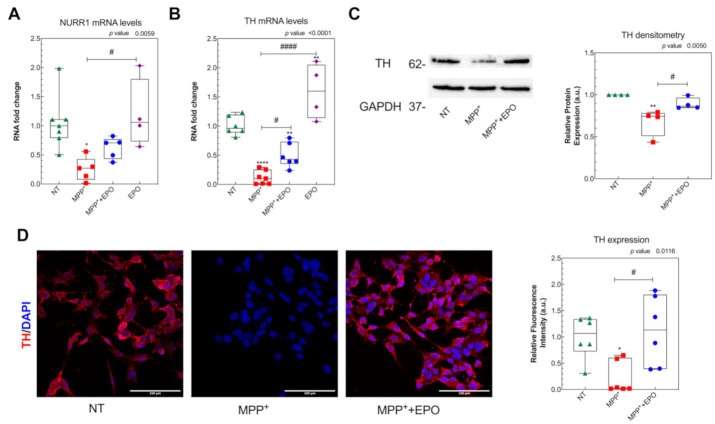
Effect of EPO on the preservation of dopaminergic targets TH and NURR1. The expression levels of NURR1 (**A**) and tyrosine hydroxylase (TH). * *p* <0.05, # *p* < 0.05 vs. MPP^+^ (**B**) mRNA were evaluated by Real Time-PCR in SH-SY5Y cells, after 24 h of treatment with MPP^+^, MPP^+^+EPO or EPO. GAPDH was used as housekeeping gene. Data are expressed as box-and-whisker plot of 3 replicate values in 3 independent experiments (*n* = 9). ** *p* < 0.01, **** *p* <0.0001 vs. NT; # *p* < 0.05, #### *p* <0.0001 vs. MPP^+^. (**C**) TH protein expression was evaluated by Western blot in SH-SY5Y cells, after 24 h of treatment with MPP^+^ or MPP^+^+EPO. The intensity of the band and relative protein expression was evaluated with the software Image-J (NIH). Data reported are presented in a box-and-whisker plot of 4 independent experiments and results are represented as intensity of TH vs. GAPDH, in relation to NT. GAPDH was used as loading control. ** *p* < 0.01 vs. NT; # *p* < 0.05 vs. MPP^+^. (**D**) TH protein expression was evaluated by immunofluorescence analysis in SH-SY5Y cells, after 24 h of treatment with MPP^+^ or MPP^+^+EPO. Fluorescence quantification and the relative protein expression were performed with the software Image-J (NIH). Scale bar: 100 µm. Data reported are presented in the form of a box-and-whisker plot (3 fields per experiment, 2 independent experiments; *n* = 6). * *p* < 0.05 vs. NT; # *p* < 0.05 vs. MPP^+^.

To investigate the effects of EPO on dopaminergic recovery, we measured both mRNA and protein expression of appropriate markers. While MPP^+^ reduced RNA expression of both NURR1 and TH, 4 U/mL EPO rescues the damage induced by MPP^+^ (Figure 2A,B). Moreover, 4 U/mL EPO restored the protein levels of TH, measured by either Western blot or immunofluorescence (Figure 2C,D and Supplementary Figure S1A), supporting the claim that EPO specifically affects dopaminergic neuroprotection [14]. Moreover, EPO-only administration does not influence NURR1 expression, whereas it induces TH mRNA but not protein expression (Figure 2A,B). 

**Figure 3:**
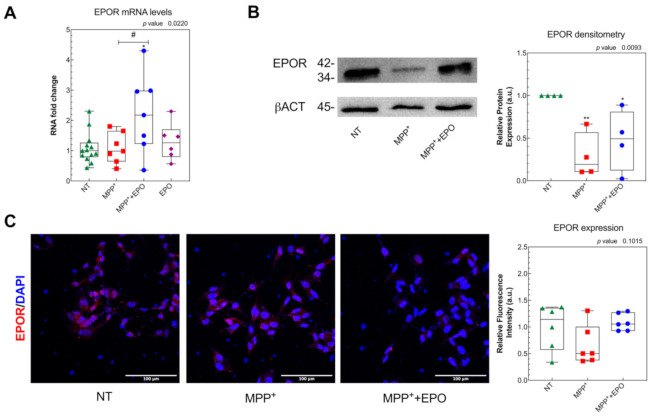
Effect of EPO on the induction of EPO receptor (EPOR) expression. (**A**) The expression levels of EPOR mRNA were evaluated by Real Time-PCR in SH-SY5Y cells, after 24 h of treatment with MPP^+^, MPP^+^+EPO or EPO. GAPDH was used as housekeeping gene. Data are expressed as mean ± SEM of 3 replicate values in 3 independent experiments (*n* = 9) and results are represented as percent of NT. * *p* < 0.05 vs. NT, # *p* < 0.05 vs. MPP^+^. (**B**) EPOR protein expression was evaluated by Western blot in SH-SY5Y cells, after 24 h of treatment with MPP^+^ or MPP^+^+EPO. GAPDH was used as loading control. The intensity of the band and relative protein expression was evaluated with the software Image-J (NIH). Data reported refer to the mean ± SEM of 4 independent experiments (*n* = 4) and they are represented as intensity of EPOR versus beta-actin, in relation to NT. * *p* < 0.05; ** *p* < 0.01 vs. NT. (**C**) EPOR protein expression was evaluated by immunofluorescence analysis in SH-SY5Y cells, after 24 h of treatment with MPP^+^ or MPP^+^+EPO. Fluorescence quantification and the relative protein expression were performed with the software Image-J (NIH). Scale bar: 100 µm. Data reported are mean ± SEM (3 fields per experiment, 2 independent experiments; *n* = 6) and results are represented as percent of NT. The grey area delimits the value (mean ± SEM) obtained in NT.

To test whether the effect of EPO is mediated by the up-regulation of EPOR expression, Figure 3 shows EPOR expression levels assayed by RT-PCR, Western blot and immunofluorescence. We found that EPOR mRNA levels were unchanged following MPP^+^ or EPO-only treatments, but they resulted upregulated in MPP^+^+4 U/mL EPO (Figure 3A). By contrast, EPOR protein expression was always downregulated when tested by Western blot and immunofluorescence analysis, with a slight non-significant increase in 4 U/mL EPO co-treatment with respect to MPP^+^ only (Figure 3B,C and Supplementary Figure S1B). Even though not statistically significant, the immunofluorescence analysis confirmed the Western blot evidence. By Western blot, we were able to discriminate and identify between different isoforms of EPOR, demonstrating that the SH-SY5Y cell line occurs with only the truncated isoform of EPOR at 37 kDa (Figure 3B; Supplementary Figure S2), as previously described by Marcuzzi and colleagues [17].

### 3.3. EPO Rescues MPP^+^-Induced Mitochondrial Dysfunction and Stimulates Glycolysis

**Figure 4:**
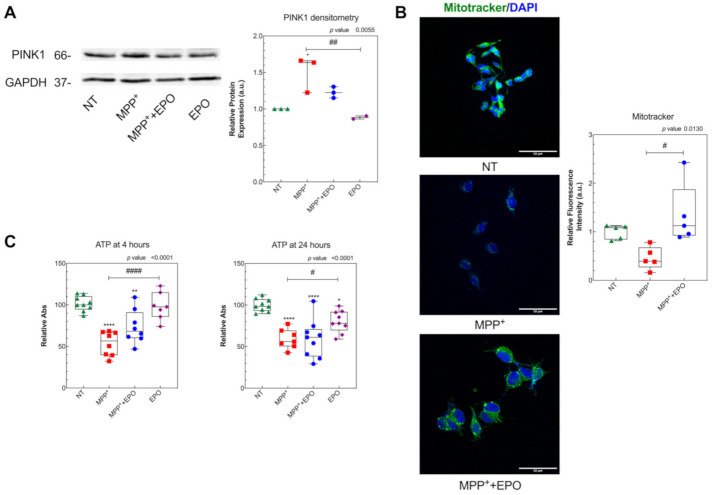
EPO counteracts MPP^+^-induced mitochondrial dysfunction: (**A**) PINK1 protein expression was evaluated by Western blot in SH-SY5Y cells, after 24 h of treatment with MPP^+^ or MPP^+^+EPO. Band intensity analysis and the relative protein expression were performed with the software Image-J (NIH). Data are presented in the form of a box-and-whisker plot of 2 independent experiments and results are represented as percent of NT. GAPDH was used as loading control. * *p* < 0.05 vs. NT; ## *p* < 0.01 vs. MPP^+^. (**B**) MitoTracker staining in SH-SY5Y cells, after 24 h of treatment with MPP^+^ or MPP^+^+EPO. MitoTracker was analyzed with immunofluorescence. Fluorescence quantification, represented in the histogram, was performed with the software Image-J (NIH). Scale bar: 50 µm. The reported data are presented in the form of a box-and-whisker plot of 3 replicate values in 2 independent experiments (*n* = 6) and results are represented as percent of NT. Results are reported as intensity of PINK1 vs. GAPDH, in relation to NT. # *p* < 0.05 vs. MPP^+^. (**C**) ATP concentration 4 h and 24 h after treatments. Data refer to 4 independent experiments and results are shown as percent of NT. * *p* < 0.05, ** *p* < 0.01, **** *p* < 0.0001 vs. NT, # *p* < 0.05, #### *p* < 0.0001 vs. MPP^+^.

To investigate EPO’s effect on mitochondrial wellness, we measured—by Western blot—the expression of PINK1, a stress-induced mitochondrial protein strictly correlated to PD [56]. We found that PINK1 expression was increased by MPP^+^ and rescued by 4 U/mL EPO (Figure 4A and Supplementary Figure S1C), with no change with the EPO-only treatment. Mitochondria were marked with the fluorescent dye Mitotracker, which accumulates into active mitochondria only, and mitochondrial viability was evaluated in living cells (Figure 4B). A non-significant decrease in mitochondrial activity was observed following MPP^+^ administration, together with a significant improvement in mitochondrial health in EPO-treated cells, as highlighted by greater fluorescence, which indicates functional recovery compared to the pathological condition (Figure 4B). Such damage by MPP^+^ and partial rescue by EPO are appreciable also by measuring the ATP level 4 h after MPP^+^ and EPO co-treatment. MPP^+^ significantly decreased the ATP level after both 4 and 24 h. EPO significantly contrasted this effect at 4 h, but not at 24 h (Figure 4C). EPO-only administration did not change the ATP levels at 4 h but slightly reduced them at 24 h (Figure 4C). 

**Figure 5:**
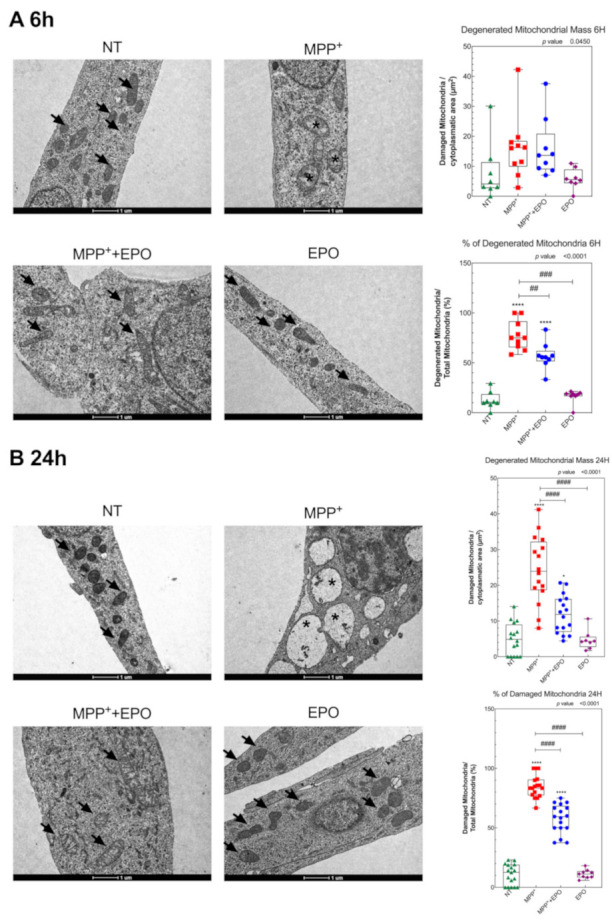
EPO promotes the preservation of mitochondria ultrastructure. Transmission electron microscopy micrographs showing a portion of cytoplasm of SH-SY5Y cells (NT), after 6 h (**A**) and 24 h **(B)** treatment with MPP^+^, MPP^+^ + EPO or EPO. Arrows indicate healthy mitochondria and asterisks indicate mitochondria with strong signs of damage (disruption of cristae architecture and reduced matrix density). Scale bars = 1 µm. Data reported in the histogram refer to the box and whisker plot of 8 area values in 1 experiment and results are represented as percent of NT. * *p* < 0.05, **** *p* < 0.0001 vs. NT; ## *p* < 0.01, ### *p* < 0.001, #### *p* < 0.0001 vs. MPP^+^.

Mitochondrial ultrastructure was investigated by TEM after 6 h or 24 h of treatment by the semi-quantitative analysis explained in the Materials and Methods section (Figure 5). The population of mitochondria with clear damage signs, e.g., cristae architecture disruption and reduced matrix density, was markedly higher in MPP^+^-treated cells, but EPO administration could partly rescue the damage (Figure 5A,B). EPO-only administration did not affect mitochondrial ultrastructure (Figure 5A,B). 

**Figure 6:**
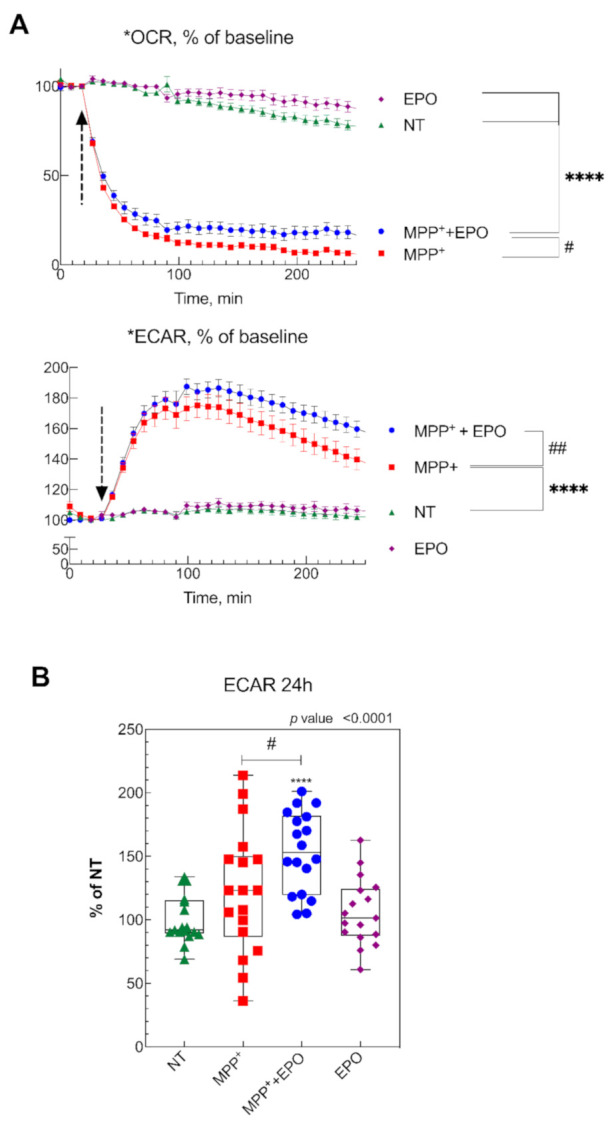
Effect of EPO on cell metabolism. (**A**) oxygen consumption rate (OCR) and extracellular acidification rate (ECAR) were measured in SH-SY5Y cells 200 min after injection of 500 µM MPP^+^ in the presence or absence of 4 U/mL EPO, or only SH-SY5Y treated with 4 U/mL. Data are represented as average of three independent experiments. **** *p* < 0.0001 vs. NT, # *p*< 0.05, ## *p* < 0.001 vs MPP^+^. (**B**) ECAR values of SH-SY5Y cells, non-treated (NT), after 24 h of treatment with MPP^+^, MPP^+^+EPO, or EPO-only. Data from 3 independent experiments per condition, normalized to the control baseline (**** *p* < 0.0001 vs. NT, # *p* < 0.05 vs. MPP^+^).

The protective effect of EPO on mitochondrial morphology was associated with changes in cellular bioenergetics. While MPP^+^ addition caused a rapid decrease in OCR, another hallmark of mitochondrial damage (Figure 6A), the treatment with EPO promoted a slight rescue in the acute phase (200 min) after treatment (Figure 6A, Supplementary Figure S3A). However, MPP^+^ also markedly increased the glycolytic rate, which was further enhanced by EPO (Figure 6A, Supplementary Figure S3A). Thus, while MPP^+^ diminishes respiration and increases the glycolytic rate, expressed in terms of the extracellular acidification rate (ECAR), EPO can counteract the deleterious effects driven by MPP^+^. We examined the effect of long-term (24 h) exposure of cells to MPP^+^ and EPO. As shown in Figure 6B, 24 h exposure of cells to MPP^+^ elicited a dramatic inhibition of the respiration. Under these conditions, the Mitostress assay revealed that MPP^+^ completely abolished the ATP-dependent mitochondrial respiration, because of lack of response following addition of oligomycin, an inhibitor of ATP synthase [48] (Supplementary Figure S3B,C). EPO-only administration did not affect OCR or ECAR (Figure 6). However, while MPP^+^ caused a rise in ECAR, EPO caused a further marked increase in ECAR (Figure 6B). This highlights that MPP^+^ induces a profound and long-lasting inhibition of mitochondrial function that is partially rescued by EPO only in the acute phase. The concomitant marked increase in the glycolytic rate in cells incubated with MPP^+^ or MPP^+^+EPO thus suggests the activation of EPO-driven robust compensatory survival and energy production mechanisms in response to mitochondrial dysfunction. 

### 3.4. EPO Promotes Functional and Dopaminergic Recovery in Parkinsonian Mice 

**Figure 7:**
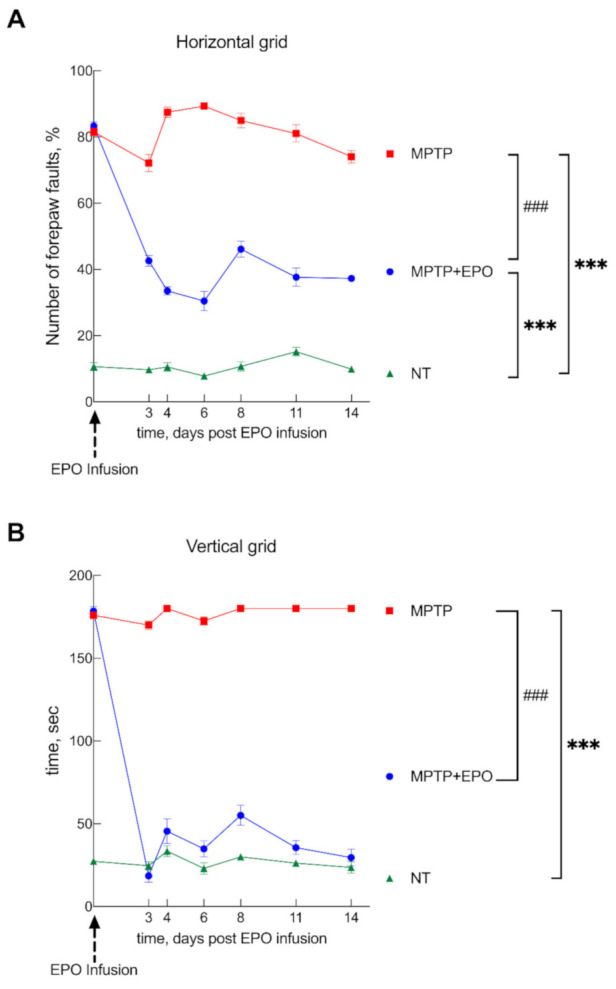
EPO promotes functional recovery in Parkinson’s disease (PD) mice. Mice were divided into three groups: healthy control, non-treated mice (NT), mice treated with MPTP to induce parkinsonism (MPTP), and mice treated with MPTP that received EPO 10 days after the induction of degeneration (day 0 see dashed arrow; MPTP + EPO). Animal behavior was monitored for 14 days after EPO administration and verified by two behavioral tests: the horizontal (**A**) and the vertical (**B**) grid test. Non-treated mice (green) are compared with MPTP (red) and MPTP+EPO (blue). Data are expressed as mean ± SEM (*n* = 6 each group). *** *p* < 0.001 vs. NT; ### *p* < 0.001 vs. MPTP.

Parkinsonism was induced in 12–15 week-old male C57BL/6J male mice by i.p. injection of MPTP at two different dosages following a described protocol [37,38,40]. As the presently reported behavioral data were obtained in a different set of animals, they represent an independent validation of previous work. Indeed, its administration markedly increases the number of forepaw faults of the anterior paw, as shown in the horizontal grid test, as well as the time to descend from the vertical grid in the specific test (Supplementary Figure S4). After intrastriatal infusion of EPO, a significant recovery of function was observed starting at day 3 after EPO administration. In the horizontal grid test, the percentage of forepaw faults in mice treated with EPO was 37.3 ± 3.1% compared to 87.4 ± 3.8% of parkinsonian animals (Figure 7A). In the vertical grid test, EPO-treated mice showed a significant improvement in the time employed to reach the base of the grid (33.5 ± 15.1 s, mean ± SD), whereas parkinsonian non-treated mice employed more than 3 min (the observation was stopped at 180.0 ± 0.2 s) (Figure 7B). The initial functional recovery observed in animals treated with injection of EPO was maintained for the entire observational period of two weeks, while MPTP mice gradually showed a decline in behavioral performance, arriving to the maximum deficit on the eighth day (Figure 7). 

**Figure 8:**
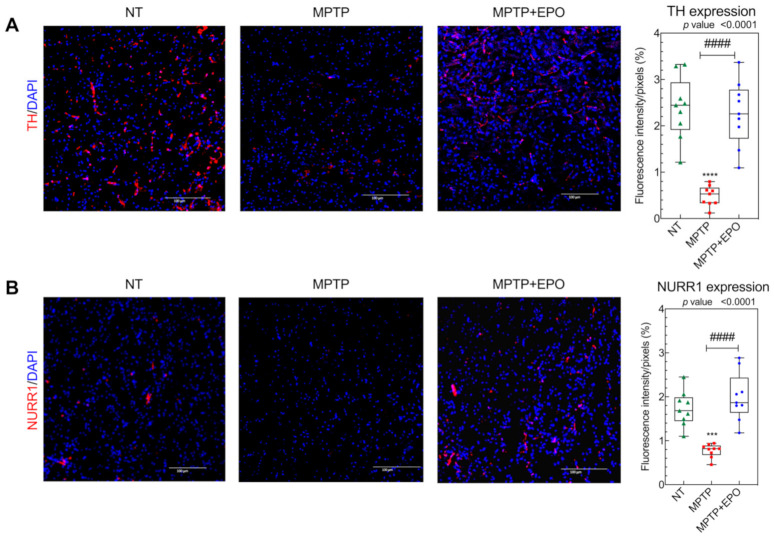
In vivo recovery of dopaminergic targets TH and NURR1. TH (**A**) and NURR1 (**B**) expression in the striatum was studied via immunofluorescence (see Section 2) in non-treated (NT), mice treated with MPTP (MPTP) and MPTP-treated mice after EPO injection (MPTP+EPO). Scale bar: 100 µm. Data for immunofluorescence quantification are expressed as box and whisker plot of 3 animals for each condition (*n* = 3 mice each condition; 3 slides per mouse; 2 images per slide) and results are represented as mean of optical density/pixels. *** *p* < 0.001 **** *p* < 0.0001 vs. NT; #### *p* < 0.0001 vs. MPTP). Fluorescence quantification was performed with the software Image-J (NIH).

We decided to investigate whether this functional recovery is due to the rescue of specific dopaminergic targets, specifically TH and NURR1 (Figure 8A,B, respectively). TH is a fundamental marker for dopamine synthesis in catecholaminergic neurons [57], whereas NURR1 is a nuclear transcription factor which regulates the expression of TH and the dopamine transporter (DAT) [58]. As expected, TH and NURR1 immunoreactivity was significantly decreased in the striatum of parkinsonian animals (Figure 8A,B) [37,38,40]. In striatum tissue sections of mice which received EPO injection, the expression of the examined markers was significantly recovered (Figure 8). Ipsilateral and contralateral striatum were analyzed, and the results showed no differences in fluorescence intensity.

**Figure 9:**
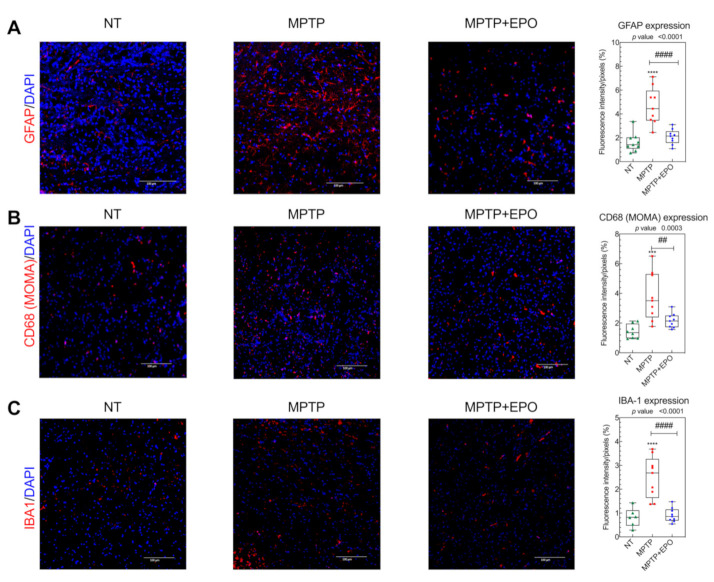
EPO counteracts the expression of neuroinflammation markers in brains of PD affected mice. anti-Glial Fibrillary Acidic Protein (GFAP) (**A**), mouse macrophage (MOMA) (**B**) and ionized calcium-binding adapter molecule 1 (IBA1) (**C**) expression in the striatum was studied via immunofluorescence (see Section 2) in non-treated mice (NT), mice treated with MPTP (MPTP), and MPTP-treated mice after EPO injection (MPTP + EPO). Scale bar: 100 µm. Data of immunofluorescence quantification are expressed as box and whisker plot of 3 animals for each condition (*n* = 3 mice each condition; 3 slides per mouse; 2 images per slide) and results are represented as mean of optical density/pixels. (*** *p* < 0.001, **** *p* < 0.0001 vs. NT; ## *p* < 0.01 vs. MPTP; #### *p* < 0.0001 vs. MPTP). Fluorescence quantification was performed with the software Image-J (NIH).

We previously demonstrated that the motor recovery in mice treated with Er-NPCs is mediated also by their anti-inflammatory and antioxidant action [40]. For this reason, we decided to investigate the effect of EPO on neuroinflammation [40]. To this end, we evaluated the expression of the acid fibrillary protein of the glia (GFAP), an astrogliosis marker (Figure 9A, Figure S5A), ionized calcium-binding adapter molecule 1 (IBA1, Figure 9B) and mouse macrophage (MOMA, Figure 9C, Figure S5B) [59]. Ipsilateral and contralateral striatum were analyzed, and the results showed no differences in fluorescence intensity. In mice treated with MPTP, we observed an increase in positivity to neuroinflammatory markers, due to the neuroinflammation caused by MPTP-mediated neurotoxic effect. By contrast, the levels of these markers were subjected to a decrease following EPO injection, likely due to the neuroprotective effect of EPO (Figure 8, Figure S5C). 

## 4. Discussion

By focusing on the protection afforded by EPO to the dopaminergic function, in this study we investigated the mechanisms underlying EPO-induced neuroprotection in vitro and in vivo. The mitochondrial redox imbalance is well known to represent a major factor responsible for mitochondrial dysfunction, especially in the pathogenesis of PD [60]. Here, we show that EPO restores cell viability in both protective and restorative layouts and contributes to the dopaminergic recovery, as shown by restored TH and NURR1 mRNA and protein levels. EPO treatment also rescues the PD-induced damage to mitochondria as shown by transmission electron microscopy, Mitotracker assay and PINK1 expression. Functional tests reveal that EPO acutely rescues the mitochondrial respiration, and markedly enhances the glycolytic rate, as shown by an increase in ECAR. These factors contribute synergistically to improve the cell ATP level. In PD mice, EPO intrastriatal infusion markedly improves the behavioral outcomes. In this study, EPO was administered in the left striatum, i.e., at the same site where Er-NPCs were injected in a previous study [38,40], in order to validate EPO’s therapeutic efficacy at the striatum level. As a matter of fact, the functional recovery is associated with molecular markers such as rescued TH and NURR1 levels, together with a reduction in neuroinflammation markers associated with PD-driven neurodegeneration.

The role of EPO is currently being investigated for its neuroprotective effects. The discovery that EPO is produced in the brain tissue, which also expresses high levels of EPOR, gave rise to a previously unknown cellular neuroplasticity mechanism that synergizes the regulation of the redox equilibrium [61,62]. Administration of rh-EPO or EPO analogs in PD rodent models has, indeed, revealed marked neuroprotective and curative effects [28,35]. In humans, two pilot studies investigated safety and efficacy of rhEPO administration. In the first, administration of 60 IU/kg bw of the Cuban rhEPO, subcutaneously once a week for five weeks in 10 PD patients improved motor function, cognitive status and mood [36]. In the second single-blind randomized trial, rh-EPO (40,000 IU each; twice a week for 5 weeks) to 13 PD patients ameliorated non-motor symptoms 12 months after administration [25]. Moreover, it has been demonstrated that a non-hematopoietic form of EPO, isolated from skimmed goat milk, is able to exert protective effects against oxidative stress [41,42].

In the in vitro part of this study, PD was recapitulated in cultured SH-SH5Y cells challenged by MPP^+^ [45]. We opted not to grow cells in the presence of retinoic acid because most literature studies using SH-SY5Y cells as a model for PD were performed in undifferentiated cells. Moreover, the cell differentiation effect exerted by retinoic acid in the context of PD has led to controversial results, because while some of them indicated increased susceptibility to dopaminergic (DAergic) neurotoxin [63], others showed decreased susceptibility to this neurotoxin without changes in DAergic markers [64]. EPO at concentrations of as low as 4 U/mL were revealed to rescue the damage induced by MPP^+^ on several cell mechanisms. Although the present study was designed for a 24 h time duration, the effect of EPO appears to persist for up to 48 h. Some experiments were designed to test whether the effects driven by EPO are attributable to neuroprotective or restorative mechanisms. Specifically, the condition where MPP^+^ and EPO are added simultaneously at t = 0 to the culture medium reflects the situation occurring when EPO is administered at the start of the pathological state, i.e., the EPO neuroprotective effect. By contrast, the condition wherein EPO is added 24 h after MPP^+^ reflects the situation occurring when EPO is administered after the onset of the pathology, i.e., the EPO curative effect. The observation that both layouts rescue the damage inferred by MPP^+^ support previous reports [24]. 

The protection afforded by EPO appears to be specific for PD. Indeed, the cell viability recovery is simultaneous with the rescue of the dopaminergic markers, TH and NURR1 [58,65], both in vitro and in vivo. Furthermore, EPO administration in vivo reduces the expression of the neuroinflammation markers GFAP, MOMA and IBA1 [4,40]. Finally, EPO injection in PD mice improves the behavioral tests, a phenomenon supported by converging results from immunohistochemical analyses.

Several studies performed in PD rodent models highlighted EPO as a possible neuroprotective agent in vivo [37,38]. EPO administration could rescue the expression of TH in rats in which parkinsonism was induced with rotenone or 6-hydroxydopamine, together with decreased levels of tumor necrosis factor alpha in the same brain areas affected by PD [32]. This suggests that EPO could exert its protective effects by contrasting neuroinflammation [37,38,66,67]. This effect has been previously proposed when investigating the effects of EPO, when released by a class of neural precursors cells in a murine model of PD with MPTP [37,38]. Recently it has been reported that EPO reduces neurodegeneration in a 6-OHDA mouse model of PD by inducing the expression of anti-apoptotic (Bcl-2) and anti-oxidant (glutathione peroxidase) factors within the striatum [68]. The same authors show a clear protective effect when EPO is administered into the striatum where it attenuates the neurodegeneration caused by 6-OHDA [68]. In agreement with such evidence, here we show that a single administration of rhEPO promotes the functional recovery by hampering the histopathological features, such as the dopaminergic loss and neuroinflammation within the striatum. Remarkably, EPO has been proposed in the treatment of severely ill SARS-CoV2 patients to improve cell respiration in the lungs, brainstem, spinal cord and respiratory muscles, to counteract the severe inflammatory state caused by the cytokine storm, and to favor neuroprotection and neuroregeneration both in the CNS and the peripheral nervous system [69,70,71]. 

As for the underlying mechanisms, the effects of EPO are likely mediated by EPO binding to the truncated isoform of EPOR, which has been identified in murine dopaminergic neurons [17]. Indeed, this isoform is expressed in SH-SY5Y cells, and its expression is markedly upregulated in EPO-treated cells [72]. 

Other potential mechanisms underlying the protective action of EPO in our model may involve the mitochondria. Several studies previously reported that EPO is involved in mitochondrial biogenesis. In an Alzheimer’s Disease in vitro model, EPO administration could counteract the oxidative stress and consequent apoptosis in PC12 cells damaged by soluble oligomers of Aβ peptide [42,73]. Furthermore, EpoL—a rhEPO variant with low glycosylation and deprived of hematopoietic effect—displays a more powerful neuroprotective profile against oxidative stress with respect to the canonical EPO isoforms in an in vitro model of PD using PC12 cells [41]. Moreover, a study on ischemic disease in non-hematopoietic cells reported increased recovery of cell function through mitochondrial activity stimulation by an endogenous EPO inducer, indicating that EPO could be a potential therapeutic strategy for ischemic diseases [74]. Since MPP^+^ is known to damage mitochondrial respiration via inhibition of complex I of the electron transport chain [75], we questioned whether EPO plays a neurotrophic role at the mitochondrial level.

To address this question, we show that EPO preserves mitochondrial morphology in cells challenged by MPP^+^. Specifically, we demonstrate that the mitochondrial uptake of Mitotracker dye is upregulated in EPO-treated cells compared to MPP^+^ only-treated cells with decreased uptake. This observation is confirmed by TEM semiquantitative analysis and by decreased PINK1 expression, which indicate reduction in the number of damaged mitochondria. The experiments reported in Figure 7 assessing cell metabolism, show that the mitochondrial respiration is only slightly restored in EPO-treated cells. It is thus likely that the protection afforded by EPO may not be completely due to rescued mitochondrial activity but may also be mediated by higher recruitment of anaerobic mechanisms. In fact, although treatment with MPP^+^ already stimulates glycolysis compared to non-EPO-treated cells, the rise in ECAR is further potentiated by EPO. As EPO partially prevents the marked reduction in ATP levels 4 h after MPP^+^ challenge, we believe that this may be attributed to an enhanced glycolytic rate that compensates for a dysfunctional respiratory chain. This unexpected finding can be related to recent observations concerning the glycolytic processes in PD pathogenesis [76,77]. Some studies indeed are focusing on the potential beneficial effects of alpha adrenergic receptors inhibitors in PD patients, such as meclizine and terazosin, that are already used to treat other diseases, for their role in enhancing the glycolytic response [77,78,79]. This observation is further enhanced by the known correlation of EPO to the glycolytic process in muscle tissue [80], and the well-characterized role of EPO during hypoxia [7,14,62,81,82,83]. The data reported here represent the first evidence of the relevance of anerobic metabolic pathways in a neurodegenerative context. Moreover, we describe for the first time how the protective effect of EPO against neurodegenerative diseases such as PD, may be ascribable to the EPO capability of restoring mitochondria metabolism and morphology. Further studies are needed to validate the role of EPO on anerobic metabolic pathways and mitochondrial health in vivo. The effects of EPO administration on the Substantia Nigra also need to be assessed, along with EPO’s efficacy when injected in other sites, such as the Substantia Nigra, or a more translatable route of administration such as intraperitoneal injection or gastric gavage.

## 5. Conclusions

In conclusion, both the in vivo and in vitro experiments reported here suggest that EPO exerts a neuroprotective effect against MPTP-induced PD and has a specific role in the recovery of dopaminergic markers. EPO appears to act through the preservation of redox equilibrium. This action is exerted both at the mitochondrial and the glycolytic process levels, with a clear improvement in cell wellness. For these reasons, treatment of Parkinson’s disease with EPO could be a potentially useful to fight oxidative stress.

## Data Availability

The data is contained within the article or Supplementary Material.

## Supplementary Materials

The following are available online at https://www.mdpi.com/2076-3921/10/1/121/s1, Figure S1: Specificity of EPO antibody. The EPOR antibody used recognizes both the 55kDa and 37kDa isoform, as shown by Western blot analysis. Figure S2: Full blots for Western Blot analysis. Figure S3: Effect of EPO on cellular metabolism. Figure S4: Parkinsonism induction by MPTP administration in mice. Figure S5: Percentage of cells positive to GFAP (**A**), MOMA (**B**) and IBA1 (**C**).

## Abbreviations

ECAR: extracellular acidification rate; EPO: Erythropoietin; EPOR: erythropoietin receptor; ErNPCs: erythropoietin releasing neural progenitor cells; GFAP: acid fibrillary protein of the glia; IBA1: ionized calcium-binding adapter molecule 1; MOMA: mouse macrophages CD68; MPP+: 1-methyl-4-phenylpyridinium; MPTP: 1-methyl-4-phenyl-1,2,3,6-tetrahydropyridine; NPCs: neural progenitor cells; NT: non- treated; NURR1: nuclear receptor related-1; OCR: oxygen consumption rate; PD: Parkinson’s disease; Rh-EPO: recombinant human erythropoietin; TEM: transmission electron microscopy; TH: tyrosine hydroxylase.

## References

[1] Tysnes O.B., Storstein A. (2017). Epidemiology of Parkinson’s disease. J. Neural. Transm. (Vienna).

[2] Subramaniam S.R., Chesselet M.F. (2013). Mitochondrial dysfunction and oxidative stress in Parkinson’s disease. Prog. Neurobiol..

[3] Umeno A., Biju V., Yoshida Y. (2017). In vivo ROS production and use of oxidative stress-derived biomarkers to detect the onset of diseases such as Alzheimer’s disease, Parkinson’s disease, and diabetes. Free Radic. Res..

[4] Whitton P.S. (2007). Inflammation as a causative factor in the aetiology of Parkinson’s disease. Br. J. Pharm..

[5] Blesa J., Trigo-Damas I., Quiroga-Varela A., Jackson-Lewis V.R. (2015). Oxidative stress and Parkinson’s disease. Front. Neuroanat..

[6] Crotty G.F., Ascherio A., Schwarzschild M.A. (2017). Targeting urate to reduce oxidative stress in Parkinson disease. Exp. Neurol..

[7] Bunn H.F. (2013). Erythropoietin. Cold Spring Harb. Perspect. Med..

[8] Jelkmann W. (2001). The role of the liver in the production of thrombopoietin compared with erythropoietin. Eur. J. Gastroenterol. Hepatol..

[9] Terraneo L., Paroni R., Bianciardi P., Giallongo T., Carelli S., Gorio A., Samaja M. (2017). Brain adaptation to hypoxia and hyperoxia in mice. Redox Biol..

[10] Carelli S., Ghilardi G., Bianciardi P., Latorre E., Rubino F., Bissi M., Di Giulio A.M., Samaja M., Gorio A. (2016). Enhanced brain release of erythropoietin, cytokines and NO during carotid clamping. Neurol. Sci..

[11] Wakhloo D., Scharkowski F., Curto Y., Javed Butt U., Bansal V., Steixner-Kumar A.A., Wustefeld L., Rajput A., Arinrad S., Zillmann M.R. (2020). Functional hypoxia drives neuroplasticity and neurogenesis via brain erythropoietin. Nat. Commun..

[12] Brines M., Cerami A. (2005). Emerging biological roles for erythropoietin in the nervous system. Nat. Rev. Neurosci..

[13] Digicaylioglu M., Bichet S., Marti H.H., Wenger R.H., Rivas L.A., Bauer C., Gassmann M. (1995). Localization of specific erythropoietin binding sites in defined areas of the mouse brain. Proc. Natl. Acad. Sci. USA.

[14] Rey F., Balsari A., Giallongo T., Ottolenghi S., Di Giulio A.M., Samaja M., Carelli S. (2019). Erythropoietin as a Neuroprotective Molecule: An Overview of Its Therapeutic Potential in Neurodegenerative Diseases. ASN Neuro.

[15] Constantinescu S.N., Ghaffari S., Lodish H.F. (1999). The Erythropoietin Receptor: Structure, Activation and Intracellular Signal Transduction. Trends Endocrinol. Metab..

[16] Brines M., Grasso G., Fiordaliso F., Sfacteria A., Ghezzi P., Fratelli M., Latini R., Xie Q.W., Smart J., Su-Rick C.J. (2004). Erythropoietin mediates tissue protection through an erythropoietin and common beta-subunit heteroreceptor. Proc. Natl. Acad. Sci. USA.

[17] Marcuzzi F., Zucchelli S., Bertuzzi M., Santoro C., Tell G., Carninci P., Gustincich S. (2016). Isoforms of the Erythropoietin receptor in dopaminergic neurons of the Substantia Nigra. J. Neurochem..

[18] Soliz J., Gassmann M., Joseph V. (2007). Soluble erythropoietin receptor is present in the mouse brain and is required for the ventilatory acclimatization to hypoxia. J. Physiol..

[19] Brines M., Cerami A. (2012). The receptor that tames the innate immune response. Mol. Med..

[20] Lombardero M., Kovacs K., Scheithauer B.W. (2011). Erythropoietin: A hormone with multiple functions. Pathobiology.

[21] Erbayraktar S., Grasso G., Sfacteria A., Xie Q.W., Coleman T., Kreilgaard M., Torup L., Sager T., Erbayraktar Z., Gokmen N. (2003). Asialoerythropoietin is a nonerythropoietic cytokine with broad neuroprotective activity in vivo. Proc. Natl. Acad. Sci. USA.

[22] Gorio A., Madaschi L., Di Stefano B., Carelli S., Di Giulio A.M., De Biasi S., Coleman T., Cerami A., Brines M. (2005). Methylprednisolone neutralizes the beneficial effects of erythropoietin in experimental spinal cord injury. Proc. Natl. Acad. Sci. USA.

[23] Heikal L., Ghezzi P., Mengozzi M., Stelmaszczuk B., Feelisch M., Ferns G.A. (2016). Erythropoietin and a nonerythropoietic peptide analog promote aortic endothelial cell repair under hypoxic conditions: Role of nitric oxide. Hypoxia (Auckl.).

[24] Jang W., Kim H.J., Li H., Jo K.D., Lee M.K., Yang H.O. (2016). The Neuroprotective Effect of Erythropoietin on Rotenone-Induced Neurotoxicity in SH-SY5Y Cells Through the Induction of Autophagy. Mol. Neurobiol..

[25] Jang W., Park J., Shin K.J., Kim J.S., Youn J., Cho J.W., Oh E., Ahn J.Y., Oh K.W., Kim H.T. (2014). Safety and efficacy of recombinant human erythropoietin treatment of non-motor symptoms in Parkinson’s disease. J. Neurol. Sci..

[26] Wu Y., Shang Y., Sun S.G., Liu R.G., Yang W.Q. (2007). Protective effect of erythropoietin against 1-methyl-4-phenylpyridinium-induced neurodegenaration in PC12 cells. Neurosci. Bull..

[27] Maiese K., Chong Z.Z., Shang Y.C., Wang S. (2012). Erythropoietin: New directions for the nervous system. Int. J. Mol. Sci..

[28] Jia Y., Mo S.J., Feng Q.Q., Zhan M.L., OuYang L.S., Chen J.C., Ma Y.X., Wu J.J., Lei W.L. (2014). EPO-dependent activation of PI3K/Akt/FoxO3a signalling mediates neuroprotection in in vitro and in vivo models of Parkinson’s disease. J. Mol. Neurosci..

[29] Gunnarson E., Song Y., Kowalewski J.M., Brismar H., Brines M., Cerami A., Andersson U., Zelenina M., Aperia A. (2009). Erythropoietin modulation of astrocyte water permeability as a component of neuroprotection. Proc. Natl. Acad. Sci. USA.

[30] Gonzalez F.F., Larpthaveesarp A., McQuillen P., Derugin N., Wendland M., Spadafora R., Ferriero D.M. (2013). Erythropoietin increases neurogenesis and oligodendrogliosis of subventricular zone precursor cells after neonatal stroke. Stroke.

[31] Bond W.S., Rex T.S. (2014). Evidence That Erythropoietin Modulates Neuroinflammation through Differential Action on Neurons, Astrocytes, and Microglia. Front. Immunol..

[32] Huang C.K., Chang Y.T., Amstislavskaya T.G., Tikhonova M.A., Lin C.L., Hung C.S., Lai T.J., Ho Y.J. (2015). Synergistic effects of ceftriaxone and erythropoietin on neuronal and behavioral deficits in an MPTP-induced animal model of Parkinson’s disease dementia. Behav. Brain Res..

[33] Genc S., Kuralay F., Genc K., Akhisaroglu M., Fadiloglu S., Yorukoglu K., Fadiloğlu M., Gure A. (2001). Erythropoietin exerts neuroprotection in 1-methyl-4-phenyl-1,2,3,6-tetrahydropyridine-treated C57/BL mice via increasing nitric oxide production. Neurosci. Lett..

[34] Qi C., Xu M., Gan J., Yang X., Wu N., Song L., Yuan W., Liu Z. (2014). Erythropoietin improves neurobehavior by reducing dopaminergic neuron loss in a 6-hydroxydopamine-induced rat model. Int. J. Mol. Med..

[35] Erbaş O., Çınar B.P., Solmaz V., Çavuşoğlu T., Ateş U. (2015). The neuroprotective effect of erythropoietin on experimental Parkinson model in rats. Neuropeptides.

[36] Pedroso I., Bringas M.L., Aguiar A., Morales L., Alvarez M., Valdés P.A., Alvarez L. (2012). Use of Cuban recombinant human erythropoietin in Parkinson’s disease treatment. Med. Rev..

[37] Carelli S., Giallongo T., Viaggi C., Gombalova Z., Latorre E., Mazza M., Vaglini F., Di Giulio A.M., Gorio A. (2016). Grafted Neural Precursors Integrate Into Mouse Striatum, Differentiate and Promote Recovery of Function Through Release of Erythropoietin in MPTP-Treated Mice. ASN Neuro.

[38] Carelli S., Giallongo T., Viaggi C., Latorre E., Gombalova Z., Raspa A., Mazza M., Vaglini F., Di Giulio A.M., Gorio A. (2017). Recovery from experimental parkinsonism by intrastriatal application of erythropoietin or EPO-releasing neural precursors. Neuropharmacology.

[39] Marfia G., Madaschi L., Marra F., Menarini M., Bottai D., Formenti A., Bellardita C., Di Giulio A.M., Carelli S., Gorio A. (2011). Adult neural precursors isolated from post mortem brain yield mostly neurons: An erythropoietin-dependent process. Neurobiol. Dis..

[40] Carelli S., Giallongo T., Gombalova Z., Rey F., Gorio M.C.F., Mazza M., Di Giulio A.M. (2018). Counteracting neuroinflammation in experimental Parkinson’s disease favors recovery of function: Effects of Er-NPCs administration. J. Neuroinflamm..

[41] Castillo C., Zaror S., Gonzalez M., Hidalgo A., Burgos C.F., Cabezas O.I., Hugues F., Jimenez S.P., Gonzalez-Horta E., Gonzalez-Chavarria I. (2018). Neuroprotective effect of a new variant of Epo nonhematopoietic against oxidative stress. Redox Biol..

[42] Castillo C., Fernández-Mendívil C., Buendia I., Saavedra P., Meza C., Parra N.C., Lopez M.G., Toledo J.R., Fuentealba J. (2019). Neuroprotective effects of EpoL against oxidative stress induced by soluble oligomers of Aβ peptide. Redox Biol..

[43] Choi M., Ko S.Y., Lee I.Y., Wang S.E., Lee S.H., Oh D.H., Kim Y.S., Son H. (2014). Carbamylated erythropoietin promotes neurite outgrowth and neuronal spine formation in association with CBP/p300. Biochem. Biophys. Res. Commun..

[44] Xiong T., Yang X., Qu Y., Chen H., Yue Y., Wang H., Zhao F., Li S., Zou R., Zhang L. (2019). Erythropoietin induces synaptogenesis and neurite repair after hypoxia ischemia-mediated brain injury in neonatal rats. Neuroreport.

[45] Xicoy H., Wieringa B., Martens G.J. (2017). The SH-SY5Y cell line in Parkinson’s disease research: A systematic review. Mol. Neurodegener..

[46] Marquez B., Zouvani I., Karagrigoriou A., Anastasiades E., Pierides A., Kyriacou K. (2003). A simplified method for measuring the thickness of glomerular basement membranes. Ultrastruct. Pathol..

[47] Giordano S., Lee J., Darley-Usmar V.M., Zhang J. (2012). Distinct effects of rotenone, 1-methyl-4-phenylpyridinium and 6-hydroxydopamine on cellular bioenergetics and cell death. PLoS ONE.

[48] Chacko B.K., Kramer P.A., Ravi S., Benavides G.A., Mitchell T., Dranka B.P., Ferrick D., Singal A.K., Ballinger S.W., Bailey S.M. (2014). The Bioenergetic Health Index: A new concept in mitochondrial translational research. Clin. Sci. (Lond.).

[49] Wilson J.L., Bouillaud F., Almeida A.S., Vieira H.L., Ouidja M.O., Dubois-Rande J.L., Foresti R., Motterlini R. (2017). Carbon monoxide reverses the metabolic adaptation of microglia cells to an inflammatory stimulus. Free Radic. Biol. Med..

[50] Cui Y.F., Hargus G., Xu J.C., Schmid J.S., Shen Y.Q., Glatzel M., Schachner M., Bernreuther C. (2010). Embryonic stem cell-derived L1 overexpressing neural aggregates enhance recovery in Parkinsonian mice. Brain.

[51] Tillerson J.L., Miller G.W. (2003). Grid performance test to measure behavioral impairment in the MPTP-treated-mouse model of parkinsonism. J. Neurosci. Methods.

[52] Kim S.T., Son H.J., Choi J.H., Ji I.J., Hwang O. (2010). Vertical grid test and modified horizontal grid test are sensitive methods for evaluating motor dysfunctions in the MPTP mouse model of Parkinson’s disease. Brain Res..

[53] Keith B.J., Franklin M.A., Paxinos G. (2008). The Mouse Brain in Stereotaxic Coordinates, Compact: The Coronal Plates and Diagrams.

[54] Bordoni M., Scarian E., Rey F., Gagliardi S., Carelli S., Pansarasa O., Cereda C. (2020). Biomaterials in Neurodegenerative Disorders: A Promising Therapeutic Approach. Int. J. Mol. Sci..

[55] Jensen E.C. (2013). Quantitative analysis of histological staining and fluorescence using ImageJ. Anat. Rec. (Hoboken).

[56] Arena G., Valente E.M. (2017). PINK1 in the limelight: Multiple functions of an eclectic protein in human health and disease. J. Pathol..

[57] Weihe E., Depboylu C., Schütz B., Schäfer M.K., Eiden L.E. (2006). Three types of tyrosine hydroxylase-positive CNS neurons distinguished by dopa decarboxylase and VMAT2 co-expression. Cell. Mol. Neurobiol..

[58] Chu Y., Le W., Kompoliti K., Jankovic J., Mufson E.J., Kordower J.H. (2006). Nurr1 in Parkinson’s disease and related disorders. J. Comp. Neurol..

[59] Parillaud V.R., Lornet G., Monnet Y., Privat A.L., Haddad A.T., Brochard V., Bekaert A., de Chanville C.B., Hirsch E.C., Combadière C. (2017). Analysis of monocyte infiltration in MPTP mice reveals that microglial CX3CR1 protects against neurotoxic over-induction of monocyte-attracting CCL2 by astrocytes. J. Neuroinflamm..

[60] Schon E.A., Przedborski S. (2011). Mitochondria: The next (neurode)generation. Neuron.

[61] Baciu I., Oprişiu C., Derevenco P., Vasile V., Mureşan A., Hriscu M., Chiş I. (2000). The brain and other sites of erythropoietin production. Rom. J. Physiol..

[62] Bartesaghi S., Marinovich M., Corsini E., Galli C.L., Viviani B. (2005). Erythropoietin: A novel neuroprotective cytokine. Neurotoxicology.

[63] Lopes F.M., Schröder R., da Frota M.L., Zanotto-Filho A., Müller C.B., Pires A.S., Meurer R.T., Colpo G.D., Gelain D.P., Kapczinski F. (2010). Comparison between proliferative and neuron-like SH-SY5Y cells as an in vitro model for Parkinson disease studies. Brain Res..

[64] Cheung Y.T., Lau W.K., Yu M.S., Lai C.S., Yeung S.C., So K.F., Chang R.C. (2009). Effects of all-trans-retinoic acid on human SH-SY5Y neuroblastoma as in vitro model in neurotoxicity research. Neurotoxicology.

[65] Nutt J.G., Wooten G.F. (2005). Clinical practice. Diagnosis and initial management of Parkinson’s disease. N. Engl. J. Med..

[66] Aalling N., Hageman I., Miskowiak K., Orlowski D., Wegener G., Wortwein G. (2018). Erythropoietin prevents the effect of chronic restraint stress on the number of hippocampal CA3c dendritic terminals-relation to expression of genes involved in synaptic plasticity, angiogenesis, inflammation, and oxidative stress in male rats. J. Neurosci. Res..

[67] Zhang X., Li Q.Y., Xiao B.G. (2012). Anti-inflammatory effect of erythropoietin therapy on experimental autoimmune encephalomyelitis. Int. J. Neurosci..

[68] Thompson A.M., Farmer K., Rowe E.M., Hayley S. (2020). Erythropoietin modulates striatal antioxidant signalling to reduce neurodegeneration in a toxicant model of Parkinson’s disease. Mol. Cell. Neurosci..

[69] Ehrenreich H., Weissenborn K., Begemann M., Busch M., Vieta E., Miskowiak K.W. (2020). Erythropoietin as candidate for supportive treatment of severe COVID-19. Mol. Med..

[70] Hadadi A., Mortezazadeh M., Kolahdouzan K., Alavian G. (2020). Does recombinant human erythropoietin administration in critically ill COVID-19 patients have miraculous therapeutic effects?. J. Med. Virol..

[71] Soliz J., Schneider-Gasser E.M., Arias-Reyes C., Aliaga-Raduan F., Poma-Machicao L., Zubieta-Calleja G., Furuya W.I., Trevizan-Baú P., Dhingra R.R., Dutschmann M. (2020). Coping with hypoxemia: Could erythropoietin (EPO) be an adjuvant treatment of COVID-19?. Respir. Physiol. Neurobiol..

[72] Held M.A., Greenfest-Allen E., Su S., Stoeckert C.J., Stokes M.P., Wojchowski D.M. (2020). Phospho-PTM proteomic discovery of novel EPO- modulated kinases and phosphatases, including PTPN18 as a positive regulator of EPOR/JAK2 Signaling. Cell Signal..

[73] Ma R., Xiong N., Huang C., Tang Q., Hu B., Xiang J., Li G. (2009). Erythropoietin protects PC12 cells from beta-amyloid(25–35)-induced apoptosis via PI3K/Akt signaling pathway. Neuropharmacology.

[74] Hsu P.L., Horng L.Y., Peng K.Y., Wu C.L., Sung H.C., Wu R.T. (2013). Activation of mitochondrial function and Hb expression in non-haematopoietic cells by an EPO inducer ameliorates ischaemic diseases in mice. Br. J. Pharm..

[75] Zhu J.H., Gusdon A.M., Cimen H., Van Houten B., Koc E., Chu C.T. (2012). Impaired mitochondrial biogenesis contributes to depletion of functional mitochondria in chronic MPP+ toxicity: Dual roles for ERK1/2. Cell Death Dis..

[76] Requejo-Aguilar R., Lopez-Fabuel I., Jimenez-Blasco D., Fernandez E., Almeida A., Bolaños J.P. (2015). DJ1 represses glycolysis and cell proliferation by transcriptionally up-regulating pink1. Biochem. J..

[77] Cai R., Zhang Y., Simmering J.E., Schultz J.L., Li Y., Fernandez-Carasa I., Consiglio A., Raya A., Polgreen P.M., Narayanan N.S. (2019). Enhancing glycolysis attenuates Parkinson’s disease progression in models and clinical databases. J. Clin. Investig..

[78] Foltynie T. (2019). Glycolysis as a therapeutic target for Parkinson’s disease. Lancet Neurol..

[79] Hong C.T., Chau K.-Y., Schapira A.H.V. (2016). Meclizine-induced enhanced glycolysis is neuroprotective in Parkinson disease cell models. Sci. Rep..

[80] Mille-Hamard L., Billat V.L., Henry E., Bonnamy B., Joly F., Benech P., Barrey E. (2012). Skeletal muscle alterations and exercise performance decrease in erythropoietin-deficient mice: A comparative study. BMC Med. Genom..

[81] Marti H.H. (2004). Erythropoietin and the hypoxic brain. J. Exp. Biol..

[82] Jelkmann W. (2016). Erythropoietin. Front. Horm. Res..

[83] Bernaudin M., Bellail A., Marti H.H., Yvon A., Vivien D., Duchatelle I., Mackenzie E.T., Petit E. (2000). Neurons and astrocytes express EPO mRNA: Oxygen-sensing mechanisms that involve the redox-state of the brain. Glia.

