# Supplementary material for: Mitochondrial Metabolism as Target of the Neuroprotective Role of Erythropoietin in Parkinson’s Disease
Source: Antioxidants (Basel). 2021 Jan 15;10(1):121. doi: 10.3390/antiox10010121 (PMC7830512; doi:10.3390/antiox10010121)
Supplement: Supplementary file 1 [file antioxidants-10-00121-s001.pdf]

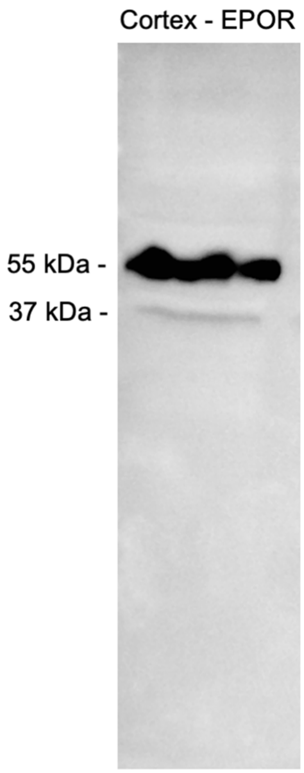

Figure S1. Specificity of EPO antibody. The EPOR antibody used recognizes both the 55kDa and 37kDa iso-form, as shown by Western blot analysis.

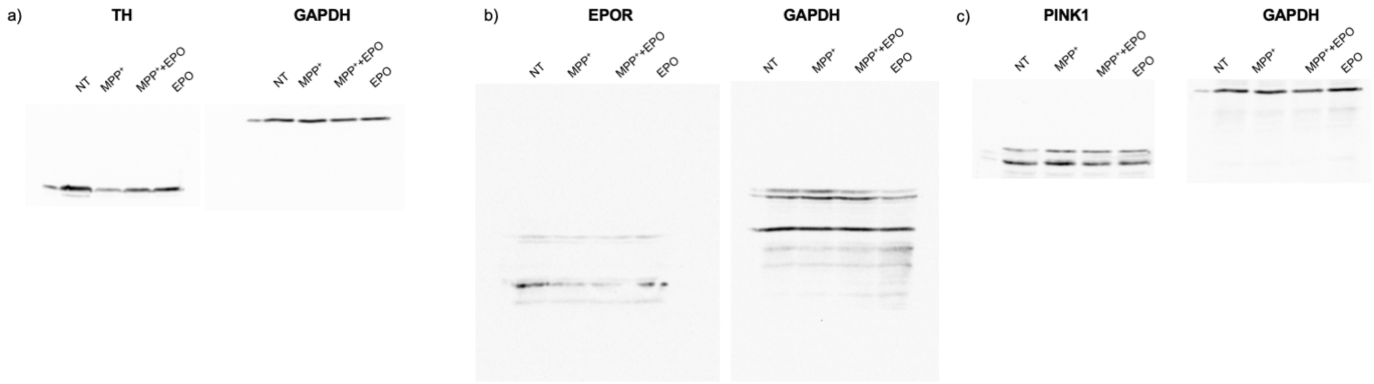

Figure S2. Full blots for Western Blot analysis. (A) TH protein expression was evaluated by Western blot in SH-SY5Y cells, after 24 h of treatment with MPP+ or MPP++EPO. GAPDH was used as house-keeping gene. (B) EPOR protein expression was evaluated by Western blot in SH-SY5Y cells, after 24 h of treatment with MPP+ or MPP++EPO. b-actin was used as housekeeping gene. (C) PINK1 protein expression was evaluated by Western blot in SH-SY5Y cells, after 24 h of treatment with MPP+ or MPP++EPO. GAPDH was used as housekeeping gene.

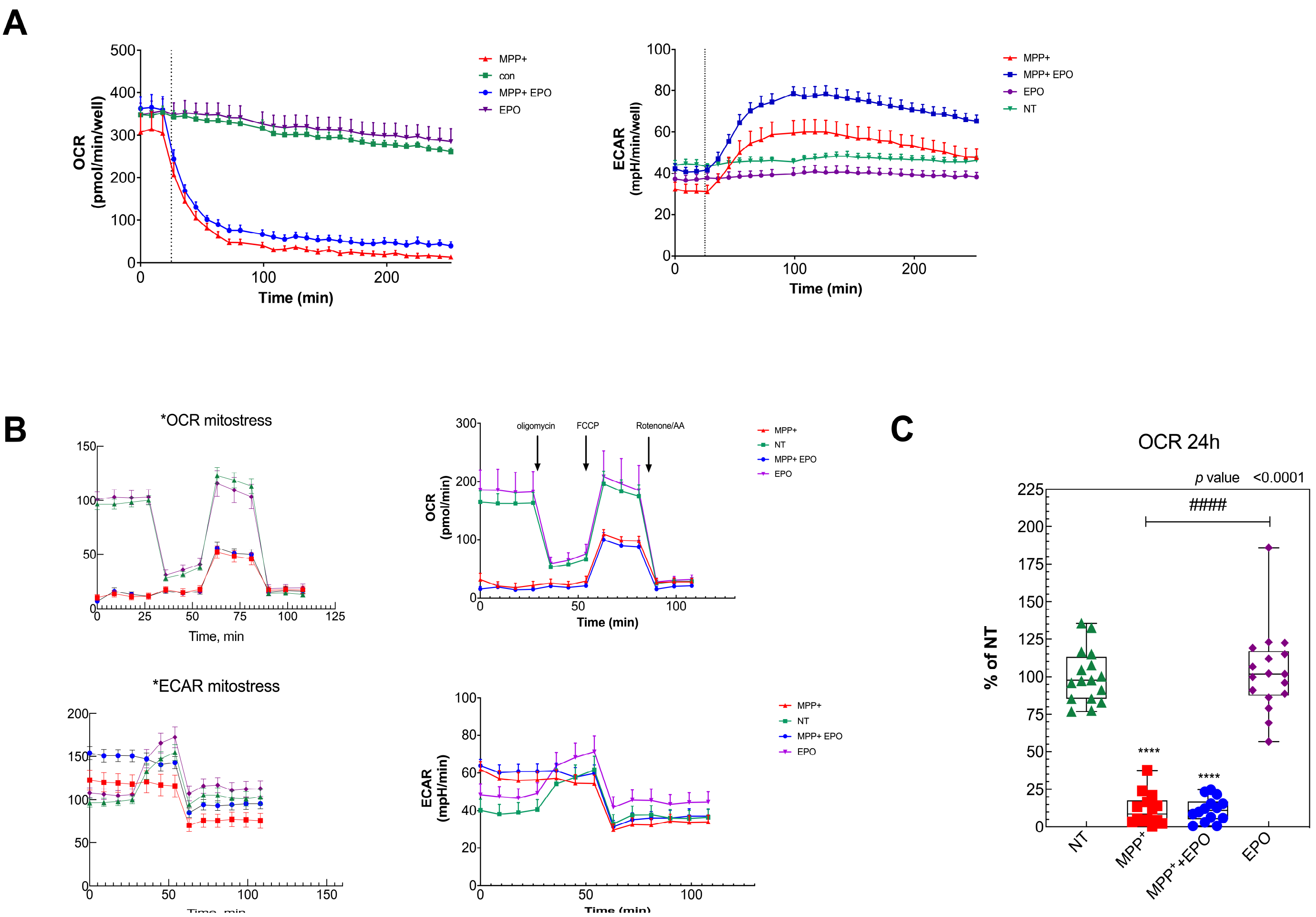

Figure S3. Effect of EPO on cellular metabolism. (A) OCR and ECAR after the MPP+ 500 μM treatment, and MPP+ plus EPO 4 U/mL co-treatment. Data from one single experiment is shown as an example of the range values normalized to the control baseline in the Figure 6A of the main text (B) OCR and ECAR values of SH-SY5Y cells, after 24 h in MPP+ 500 μM treatment, and MPP+ plus EPO 4 U/mL co-treatment. A Mitostress was performed through the sequential injection of Oligomycin, FCCP and Rotenone/Antimycin. Data from three independent experiments per condition, normalized to the control baseline ($p < 0.05$ vs. NT, # p <0.05 vs. MPP+), and from one single experiment as an example of the range values normalized to the control baseline. (C) OCR values of SH-SY5Y cells, after 24 h in MPP+ 500 μM treatment, and MPP+ plus EPO 4 U/mL co-treatment. Data from three independent experiments per condition, normalized to the control baseline (p<0.05 vs. NT, # p < 0.05 vs. MPP+).

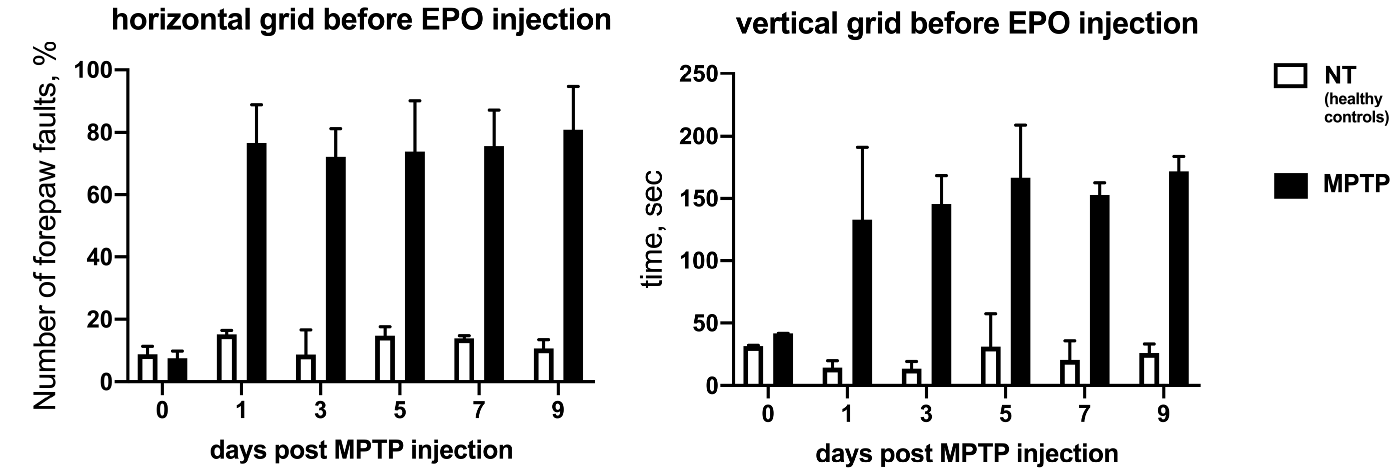

Figure S4. Parkinsonism induction by MPTP administration in mice. 12 weeks old C57BL/6J mice were subjected to intraperitoneal injection of MPTP. Motor dysfunctions were evaluated every 2 days by horizontal and vertical grid tests (see Material and Methods).

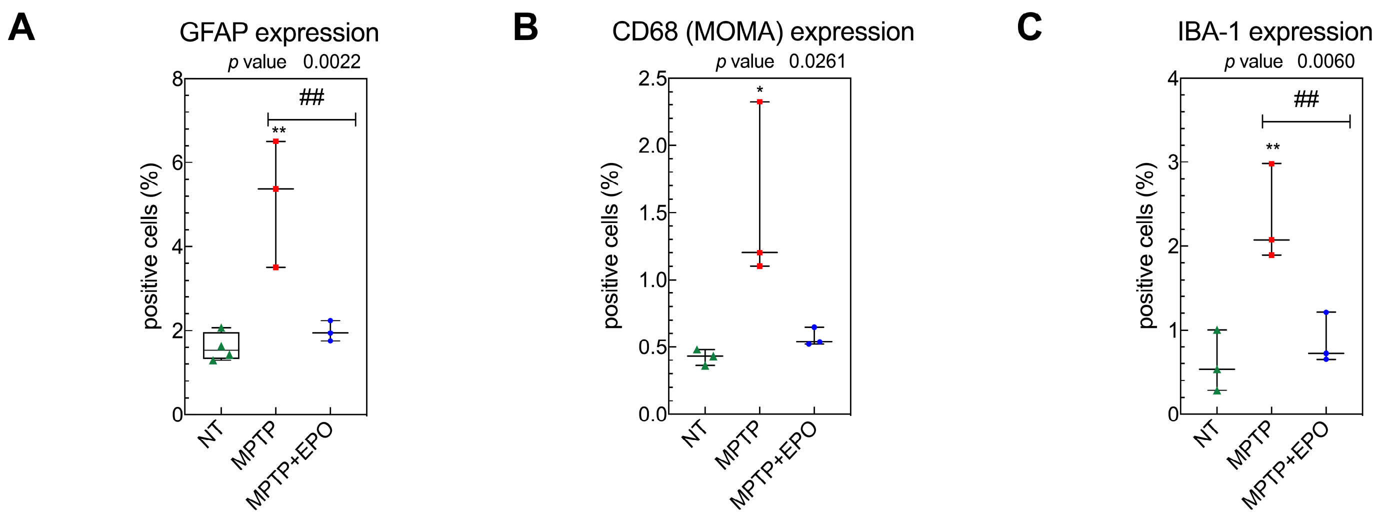

Figure S5. Percentage of cells positive to GFAP, MOMA and IBA1. The expression was studied into the striatum via immunofluorescence (see Materials and Methods) in non-treated mice (NT), mice treated with MPTP (MPTP), and MPTP-treated mice after EPO injection (MPTP+EPO). Quantification was performed as reported in Materials and Methods. Data are expressed as box and whisker plot of three animals for each condition (three slides per mouse; three images per slide) and results are represented as percent of NT. ** $p < 0.01$ vs. NT; ## $p < 0.01$ vs. MPTP. Table S1: List of primers used.
